# Clinicians’ Knowledge and Attitudes About Delirium Detection and Management

**DOI:** 10.1093/geroni/igaa057.1676

**Published:** 2020-12-16

**Authors:** Priyanka Shrestha, Erica Husser, Diane Berish, Long Ngo, Marie Boltz, Sharon Inouye, Edward Marcantonio, Donna Fick

**Affiliations:** 1 Penn State University, State College, Pennsylvania, United States; 2 Penn State, Spring Mills, Pennsylvania, United States; 3 Pennsylvania State University, University Park, Pennsylvania, United States; 4 Beth Israel Deaconess Medical Center, Boston, Massachusetts, United States; 5 Penn State University, University Park, Pennsylvania, United States; 6 Harvard University, Boston, Massachusetts, United States; 7 Penn State, University Park, Pennsylvania, United States

## Abstract

Delirium is a serious and potentially life-threatening problem, but it remains clinically under-recognized. Various factors contribute to this under-recognition, including limited understanding of delirium, insufficient training and application of delirium assessments, potential stigma for the patient and increased workload for the clinician. As a part of an NIH funded study testing a rapid two-step delirium identification protocol at two hospitals in the U.S. (one urban and one rural), clinicians completed a 12-item survey to assess their knowledge and attitudes about delirium and their confidence in preventing and managing delirium. Survey response options followed a 5-point rating scale (strongly disagree, disagree, undecided, agree, strongly agree). The sample for this analysis included 399 clinicians (MDs=53; RNs=235; CNAs=111). Chi-square was used to test for group differences between clinician types. Less than half of the clinicians reported agreeing with the statement, “delirium is largely preventable” (MDs: 47%; RN: 44%; CNA: 41%, p-value=0.021). MDs and RNs indicated a high level of confidence in recognizing delirium while CNAs endorsed lower levels of confidence (MDs: 87%; RN: 81%; CNA: 65%, p-value=0.001). All types of clinicians reported lower confidence in managing delirium (MDs: 29%; RN: 36%; CNA: 44%, p-value=0.117). 47% of CNAs and 37% of RNs agreed there is a need for additional training in caring for persons with delirium while only 21% of MDs agreed (p = 0.031). Understanding how different types of clinicians think and feel about delirium will inform training and communication initiatives, clinical implementation, and research on best practices for delirium identification and management.

